# Editorial: Insights in pediatric critical care 2022

**DOI:** 10.3389/fped.2023.1245772

**Published:** 2023-07-11

**Authors:** Nicole Shilkofski, Niranjan Kissoon

**Affiliations:** ^1^ Departments of Pediatrics and Anesthesiology/Critical Care Medicine, Johns Hopkins University School of Medicine, Baltimore, MD, United States; ^2^ Department of Pediatrics and Emergency Medicine, University of British Columbia, Vancouver, BC, Canada

**Keywords:** pediatric critical care, globalization of medicine, insights and advancements, pediatric ICU, global burden of disease

**Editorial on the Research Topic** Insights in pediatric critical care 2022

“I am not an Athenian or a Greek, but a citizen of the world”. Socrates

Under 5 deaths contribute disproportionately to critical illness and global deaths (1). The reason for this disproportionate contribution are multifactorial, however a major contributor is inequities in access to care for the critically ill resulting in many preventable deaths. In recent years as socioeconomic status have improved, pediatric critical care is becoming more accessible and colleagues across the world are sharing their knowledge and expertise and building communities of practice.

The articles in this collection demonstrate the increasing globalization of pediatric critical care medicine, representing authors of case reports, a systematic review and original research manuscripts spanning 3 different continents, including Asia, North America and Europe/United Kingdom. While healthcare resources vary from country to country, studies that permit international comparisons are needed to better understand how differences in critical care resources, organization, and delivery influence patient outcomes (2). As we begin to emerge from the SARS CoV-2 pandemic, we continue to see the disease profile in pediatric critical care medicine evolve and change, but also to realize that knowledge- sharing across continents and cultures is critical to understanding that evolution.

Optimal care of the critically ill child is best achieved by a holistic approach that goes beyond correcting or reversing abnormal physiology. This collection nicely encompasses the vast spectrum of high quality research being performed in pediatric critical care medicine, ranging from improvements in clinical care of critically ill children including the search for biomarkers to predict mortality, to addressing communication during end of life care, to implementation science approaches to improve clinical care decisions, to understanding the spectrum of available online educational interventions. This emphasizes the multifaceted scope of practice that pediatric critical care medicine entails, and we hope gives readers insight into some of the current “hot topics” worthy of further investigation and future study within this field.

In the article by Carreras et al., the authors demonstrate validity of their hypothesis that non-thyroidal illness syndrome (NTIS), as defined by low levels of free thyroxine and free triiodothyronine hormones, is associated with increased prediction of mortality risk scores in a prospective observational study of 103 patients in a pediatric intensive care unit (PICU) in Spain. While Pediatric Risk of Mortality (PRISM) scores have traditionally been utilized to improve prognostic assessment of critically ill children in the first several hours after admission to the PICU, this study is one example of the recent push to improve this prognostic assessment through the use of biochemical testing that can be quickly obtained on admission and improve accuracy of predictive models. While further studies are needed to determine if NTIS is an independent predictor of mortality, the study highlights a potential systematic maladaptive response to critical illness that could impact initial evaluation and subsequent treatment of critically ill pediatric patients. Similarly, the study by Zhou et al. sought to determine if serum phosphate levels before and during continuous renal replacement therapy (CRRT) are predictive of higher 90 day mortality rates in a population of critically ill children in a PICU in China. The correlation of hyperphosphatemia with higher mortality rates in this study points to yet another important potential biomarker for risk stratification of this subpopulation of children requiring CRRT.

The article by Resch et al. highlights use of hirudotherapy (medicinal leech therapy) in a retrospective pediatric case series of patients hospitalized in a PICU in the United States with acute refractory limb ischemia from arterial malperfusion. While the use of the enzymatic properties of leeches is not a new therapeutic modality, it is not a widely used therapy, despite being approved by the U.S. Food and Drug Administration for improving venous congestion in graft tissue. Being a case series, however, the authors conclude that further prospective studies, including consideration of bleeding manifestations as a side effect of therapy, are required prior to a systematic recommendation of hirudotherapy for use in pediatric ICUs.

Reuland et al. describe the use of a Systems Engineering Initiative for Patient Safety (SEIPS) framework within implementation science for the initiation of a Pediatric Early Warning Score (PEWS) system in the limited-resource setting of a pediatric hospital in the Philippines. To date, PEWS systems have been used predominantly in resource-rich settings to identify children at risk of acute clinical deterioration and to make decisions about care escalation in these environments, but have not been extensively studied in low and middle income countries (LMICs). This type of qualitative research to examine barriers and facilitators that exist in a resource-constrained environment is a critical predecessor to successful adaptation and implementation of PEWS in this context.

The case report by Santos et al. describes two children with encephalitis secondary to parainfluenza and respiratory syncytial virus (RSV) complicated by cytokine storm and multiorgan inflammatory response. These cases highlight that further investigations are clearly needed to better understand the pathophysiologic mechanisms of brain injury and systemic inflammatory response in critically ill children.

The EVOLvE study by Zanin et al. is a cross-sectional observational study surveying 198 pediatric critical care professionals across different regions in Europe regarding their attitudes toward end of life (EOL) care and EOL decisions. Importantly, the study highlights differences in opinions and practices from healthcare professionals regarding optimal timing of EOL decisions and underscores the need to identify and understand cultural, religious, legal and resource differences that may be the basis for discrepancies in practices and attitudes toward EOL care.

Finally, the systematic review by Daniel and Wolbrink examines the current state of evidence for online education targeting healthcare workers in PICUs. Given the widespread and sudden need for medical education to make an unprecedented pivot to online education as a primary modality during the SARS CoV-2 pandemic, this review brings timely and salient insights. The authors’ conclusion that significant opportunities remain to assess impact of online educational interventions, particularly those related to patient outcomes, should be a call to action for the pediatric critical care community, in particular individuals invested in furthering the quality of medical education in pediatrics.

Much needs to be done to improve outcomes for critically ill children globally. The sharing of knowledge is important to understand contributors to and methods to improve the outcomes for children globally, and this collection represents this effort.
